# Comparing the effects of decreasing prescription opioid shipments and the release of an abuse deterrent OxyContin formulation on opioid overdose fatalities in WV: an interrupted time series study

**DOI:** 10.1186/s13011-023-00587-2

**Published:** 2024-01-04

**Authors:** Eric W. Lundstrom, Zheng Dai, Caroline P. Groth, Brian Hendricks, Erin L. Winstanley, Marie Abate, Gordon S. Smith

**Affiliations:** 1 Department of Epidemiology and Biostatistics, School of Public Health, West Virginia University, 64 Medical Center Dr, P.O. Box 9190, Morgantown, WV 26506 US; 2 Health Affairs Institute, Health Sciences Center, West Virginia University, 405 Capitol Street, Suite 514, Charleston, WV 25301 US; 3 Department of Behavioral Medicine & Psychiatry, School of Medicine, West Virginia University, 930 Chestnut Ridge Rd, Morgantown, WV 26505 US; 4 School of Pharmacy, West Virginia University, 64 Medical Center Drive, P.O. Box 9500, Morgantown, WV 26506-9500 US

**Keywords:** Opioids, Overdose, Abuse deterrent formulation, Interrupted Time Series Analysis, West Virginia

## Abstract

**Introduction:**

The 2010 release of an abuse deterrent formulation (ADF) of OxyContin, a brand name prescription opioid, has been cited as a major driver for the reduction in prescription drug misuse and the associated increasing illicit opioid use and overdose rates. However, studies of this topic often do not account for changes in supplies of other prescription opioids that were widely prescribed before and after the ADF OxyContin release, including generic oxycodone formulations and hydrocodone. We therefore sought to compare the impact of the ADF OxyContin release to that of decreasing prescription opioid supplies in West Virginia (WV).

**Methods:**

Opioid tablet shipment and overdose data were extracted from *The Washington Post* ARCOS (2006–2014) and the WV Forensic Drug Database (2005–2020), respectively. Locally estimated scatterplot smoothing (LOESS) was used to estimate the point when shipments of prescription opioids to WV began decreasing, measured via dosage units and morphine milligram equivalents (MMEs). Interrupted time series analysis (ITSA) was used to compare the impact LOESS-identified prescription supply changes and the ADF OxyContin release had on prescription (oxycodone and hydrocodone) and illicit (heroin, fentanyl, and fentanyl analogues) opioid overdose deaths in WV. Model fit was compared using Akaike Information Criteria (AIC).

**Results:**

The majority of opioid tablets shipped to WV from 2006 to 2014 were generic oxycodone or hydrocodone, not OxyContin. After accounting for a 6-month lag from ITSA models using the LOESS-identified change in prescription opioid shipments measured via dosage units (2011 Q3) resulted in the lowest AIC for both prescription (AIC = -188.6) and illicit opioid-involved overdoses (AIC = -189.4), indicating this intervention start date resulted in the preferred model. The second lowest AIC was for models using the ADF OxyContin release as an intervention start date.

**Discussion:**

We found that illicit opioid overdoses in WV began increasing closer to when prescription opioid shipments to the state began decreasing, not when the ADF OxyContin release occurred. Similarly, the majority of opioid tablets shipped to the state for 2006–2014 were generic oxycodone or hydrocodone. This may indicate that diminishing prescription supplies had a larger impact on opioid overdose patterns than the ADF OxyContin release in WV.

**Supplementary Information:**

The online version contains supplementary material available at 10.1186/s13011-023-00587-2.

## Background

Opioid-involved overdose deaths are a major public health problem in the United States (US), with more than 644,000 fatal overdoses occurring from 1999 to 2021 [1]. The opioid epidemic has been characterized by multiple waves of overdoses associated with different drug classes and routes of administration [2, 3]. The first wave was associated with overdoses due to prescription opioid medications, such as oxycodone and its brand name extended release (ER) formulation OxyContin [4]. Beginning in the late 1990’s, these and other prescription opioids were prescribed and dispensed at increasing rates throughout the US. As a result, fatal overdoses of prescription opioids increased in tandem [2, 5].

Actions taken to decrease rates of prescription medication diversion, misuse, and overdose included efforts to reduce opioid prescribing rates [6] and prescription reformulations aiming to restrict injecting or snorting tablets, including the August 2010 release of an “abuse-deterrent formulation” (ADF) of OxyContin [7]. These targeted measures largely succeeded in decreasing rates of opioid overdoses involving prescribed opioids. However, they also may have had the unintended consequence of diverting those suffering from untreated opioid use disorder (OUD) from prescription opioids to cheaper and more available dangerous illicit alternatives, such as heroin and fentanyl [2, 8].

The ADF OxyContin release in August 2010 was widely promoted as the solution to diversion and misuse of prescription opioids. Immediately following the ADF release, sales of the non-ADF OxyContin brand ceased and OxyContin was solely prescribed in the ADF formulation [9]. Previously published research suggests that this formulation change was one reason that individuals transitioned from prescription to illicit opioid use [10, 11] and that it was the primary reason for an uptick in subsequent heroin use [12]. This hypothesis is supported by decreasing rates of OxyContin misuse and overdose after the ADF release [11]. However, a recent analysis suggests that most individuals misusing OxyContin simply switched to generic ER oxycodone following the ADF release and that falling rates of generic oxycodone prescriptions were more predictive (in comparison to the ADF OxyContin release) of subsequent increases in illicit opioid overdose at the state level [13]. Moreover, some previous studies on this topic have not assessed supplies of commonly prescribed opioids such as hydrocodone in their analyses [14], an opioid which was prescribed and misused at rates comparable to OxyContin before its ADF release [15–17].

Given the importance of supply side drivers of overdose [2], omitting widely prescribed opioids from analyses limits current understanding of the transition between prescription and illicit opioid overdose in the US. Thus, using fatal opioid overdose data from West Virginia (WV), we examined the impact of decreasing opioid shipments to WV on the transition from prescription to illicit opioid overdoses in the state. However, no fixed date for the start of declining shipments was available and current time series methods require a fixed starting point. Using a data-driven approach, we used locally estimated scatterplot smoothing (LOESS) regression to identify an approximate start date for the decline in oxycodone and hydrocodone tablet shipments, measured both via dosage units (tablets) and morphine milligram equivalents (MMEs), to WV given naturally occurring quarterly variation in data. These points were used to inform an interrupted time series analysis (ITSA) of fatal prescription and illicit opioid overdoses in the state. ITSA assesses the impact of public health events by quantifying trends before and after an intervention with a known start date [18]. We also assessed the impact of the August 2010 ADF OxyContin release for comparison to our LOESS-informed analysis. In addition to elucidating the role of prescription opioid supply changes in the transition from prescription to illicit opioid overdoses in WV, this study provides a plausible framework for using ITSA to assess the impact of public health events with no intervention start date.

## Methods

### Data sources

Data on opioid prescription shipments to WV for 2006–2014 were obtained from a subset of the Drug Enforcement Agency’s (DEA) Automation of Reports and Consolidated Orders System (ARCOS) data publicly available through *The Washington Post* [19]. ARCOS tracks the flow of all schedule I/II and select schedule III/IV substances through their manufacture and subsequent distribution to points of dispersion (i.e., retail pharmacies, hospitals, practitioners, etc.). *The Washington Post* ARCOS subset contains data on individual oxycodone and hydrocodone tablet shipments or dosage units, including the addresses of each shipment manufacturer/distributor and recipient, date of shipment, number of dosages (i.e., tablets) in each shipment, strength of each dose in milligrams, and morphine milligram equivalents (MME) conversion factors for each shipment (1 for hydrocodone, 1.5 for oxycodone). While this dataset only contains information on oxycodone and hydrocodone tablet shipments, *The Washington Post* reports that the prescription opioids excluded were shipped and diverted for misuse in much smaller quantities throughout the period reported [20]; this is congruent with another published research finding that the majority of dispensed opioids are oxycodone or hydrocodone at the state level [21]. Quarterly dosage units were calculated using an ARCOS dataset column corresponding to the number of tablets in each shipment, while quarterly MMEs were calculated using the formula MME = Quantity × Strength × Conversion Factor (Supplemental Table 1) [21–23].

WV opioid-involved overdose death data for 2005–2020 were obtained from a forensic drug database (FDD) maintained at West Virginia University through an agreement with the WV Office of the Chief Medical Examiner (OCME). The WV OCME uses a state-level, centralized death investigation system which includes comprehensive drug screenings and toxicology testing for suspected drug deaths [24]. Counts of opioid-involved overdose deaths from January 2005 to December 2020 were aggregated to the quarterly level for drug-related deaths involving prescription (oxycodone or hydrocodone) or illicit opioids (heroin or a synthetic opioid other than methadone, including fentanyl, fentanyl analogs, 4-anpp, and u-47,700). While overdoses involving hydrocodone or oxycodone often occur in individuals who obtained them illicitly (i.e., through diversion), these are labeled as “prescription” here as at the time these tablets are manufactured licitly for prescription purposes in the US. Similarly, although some licitly-manufactured fentanyl is diverted, the majority of fentanyl involved in synthetic opioid overdoses is illicitly manufactured outside of the US, particularly after 2013 [25–27]. Data from WV has shown that from 2015 to 2017, only 1.7% of decedents in whom fentanyl was involved in the deaths had a prescription for the drug, compared to almost 24% of the fentanyl related deaths from 2005 to 2014 [28]. Proportions of deaths involving prescription or illicit opioids were calculated by dividing the quarterly aggregate of either category by the total number opioid-involved deaths in each quarter.

### Statistical analysis

All statistical analyses were performed in RStudio version 4.2.2 [29]. ITSA was used to assess the impact of decreasing opioid prescription shipments to WV on the proportion of opioid overdose deaths associated with prescription and illicit opioids [18, 30]. ITSA is a robust statistical approach in which the impact of an intervention is measured using segmented linear regression. Three potential interventions were investigated. First, two interventions denoted peak tablet shipments to WV, measured via both dosage units and MMEs. As shipments varied by quarter with no defined point when the decline began, we identified a plausible decline start point using LOESS of quarterly ARCOS data. LOESS fits weighted least squared regression to data in several independent variable intervals and requires no global function, providing clear graphical representations of non-linear relationships. As a result, LOESS is often used to identify inflection points (i.e., changes in slope) in non-linear data [31, 32], including in time series data of opioid prescriptions [33] and overdoses [34]. Next, an intervention for the introduction of the ADF OxyContin (released in August 2010 or 2010 Q3) was informed by previous literature; non-ADF OxyContin prescriptions ceased the same month that the ADF was released [9]. As there is likely a temporal lag between changes in prescription opioid shipments and related variations in opioid overdose rates, we lagged each intervention by transition periods of three and six months (i.e., one and two quarters, respectively) and tested each lag/transition period in separate models. This approach has been used in previous time series studies of prescription opioid supply changes during this timeframe [35–38].

An ITSA intervention may be modeled using the equation:

$${y_t} = {\beta _0} + {\beta _1}t + {\beta _2}P + {\beta _3}D + \varepsilon$$

where *y*_*t*_ is an outcome of interest (e.g. quarterly proportion of opioid overdose deaths associated with prescription or illicit opioids), ${\beta }_{0}$ is the model intercept, *t* is time, $P$ is a variable representing time since the intervention (zero before the intervention, slope of one afterwards), and $D$ is a dummy variable representing the immediate effect of the intervention [39, 40]. ${\beta }_{1}$, ${\beta }_{2}$, and ${\beta }_{3}$ represent the pre-intervention slope, the sustained post-intervention effect (i.e., a slope change impact known as a “ramp” variable), and the immediate post-intervention effect of an intervention (i.e., a “step-change” impact), respectively. Finally, error is denoted by $?$ and may include autoregressive integrated moving average (ARIMA) terms when data violate the linear regression assumption of data independence (i.e., the data is serially correlated). ARIMA models include lagged values of a time series’ dependent variable and/or its error terms and is recommended for use in ITSA when data are not independent [18, 41]. ARIMA terms were fit to opioid overdose data via inspecting autocorrelation and partial autocorrelation plots. ITSA and ARIMA parameters for each intervention were included or excluded based on the need for control of serial correlation, preservation of model parsimony, and minimization of Akaike Information Criteria (AIC) [30, 42]. Since lagged intervention points were included to account for slow changes in prescription drug supplies, and the subsequent impact on fatal overdose would be expected to be gradual rather than immediate on a statewide scale, step change (i.e., immediate impact) variables were not included [41]; measuring slope changes only is an approach similar to published research on this topic [7, 14]. Final ITSA models were assessed for proper fit via inspection of each model’s ACF and PACF plots, as well as inspecting the significance of each model’s Ljung-Box statistic (with a non-significant value considered a properly fitting model) [43]. The AIC of each final ITSA model was used for model comparison. To compare models using AIC, we abided by the convention that when comparing two models, the model with lower AIC is better fit and that a difference of two or more AIC units is meaningful [44].

## Results

**Fig. 1:**
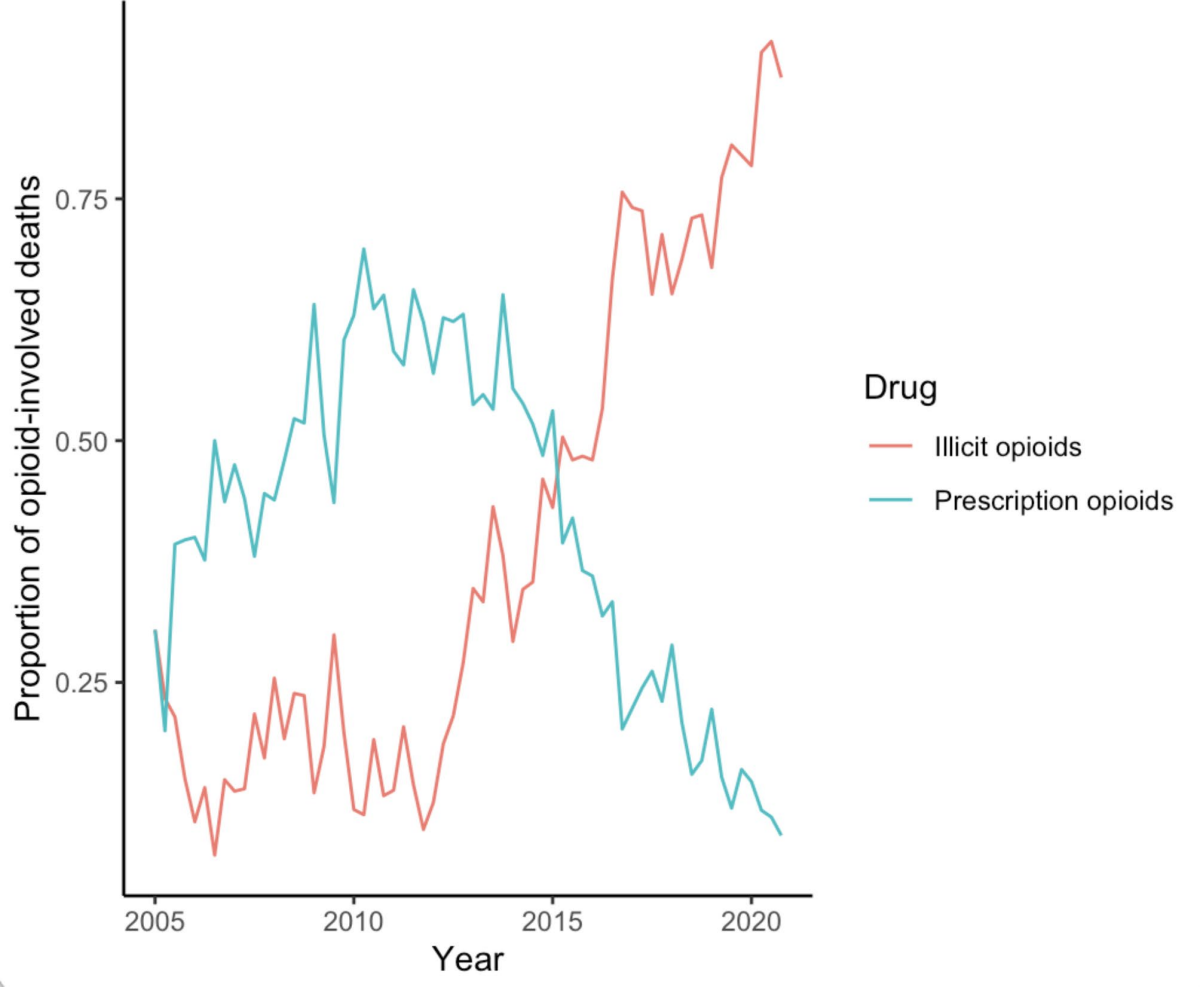
The quarterly proportion of opioid overdoses in WV associated with prescription and illicit opioids. ^a^ Prescription opioid overdoses were defined as those associated with oxycodone or hydrocodone, while illicit overdoses were defined as those involving heroin and synthetic opioids other than methadone, including fentanyl, fentanyl analogues, 4-anpp, and u-47,700. Data from the West Virginia Forensic Drug Database, which compiles data from the West Virginia Office of the Chief Medical Examiner

From 2005 to 2020, a total of 9419 opioid-involved overdose deaths were identified in the WV FDD. The proportion of deaths involving illicit opioids (synthetic opioids including fentanyl or heroin) was 0.48, while the proportion involving prescription opioids (oxycodone or hydrocodone) was 0.37; the majority of remaining fatal overdoses involved methadone (data not shown). Graphical representation of the quarterly proportion of opioid-involved overdose deaths involving prescription or illicit opioids is presented in Fig. 1. During the first two quarters of the study period, illicit opioid overdoses occurred at a rate comparable to prescription opioids; this is potentially related to a multi-state fentanyl overdose outbreak that occurred from 2005 to 2007 [45]. After this, prescription opioid overdoses occurred at a rate greater than those associated with illicit opioids until approximately 2015; at this time (2015 and later), overdose patterns changed substantially, with the majority of overdoses being associated with illicit opioids.

**Fig. 2:**
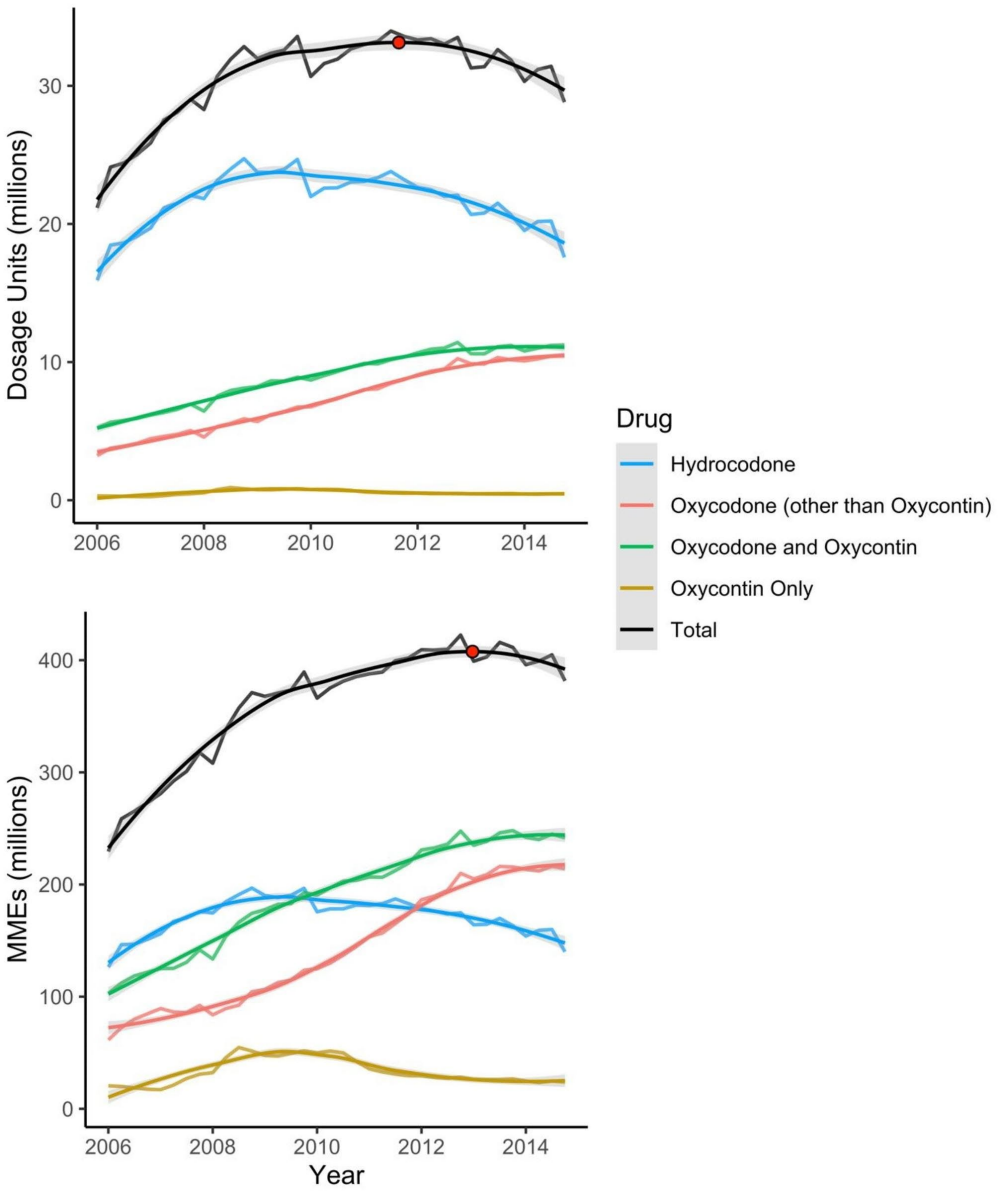
Quarterly opioid tablet shipments to West Virginia, both total and by individual opioid product and estimated change point in total opioid shipments indicated by red dot. ^a^ Data are presented measured via dosage units and morphine milligram equivalents (MMEs) and are smoothed using locally estimated scatterplot smoothing (LOESS) regression to allow for the visualization of overall trends. Peak total quarterly dosage units and MMEs were identified via LOESS and are denoted using a red dot. Data are form the Drug Enforcement Agency’s Automation of Reports and Consolidated Orders System (ARCOS) database and were obtained from The Washington Post

LOESS regression of 2006–2014 ARCOS data indicated that maximum quarterly shipments of oxycodone and hydrocodone tablets occurred in 2011 Q3 when measured via dosage units (i.e., number of tablets) and 2012 Q4 when measured via MMEs. Graphical representation of quarterly dosage units and MMEs are presented in Fig. 2 along with estimated peak total prescription shipments. From their peaks to the end of the available ARCOS data (2014 Q4), dosage unit and MME shipments decreased 15.2% and 9.7%, respectively. Measured via dosage units, hydrocodone was shipped to WV in highest quantities, followed by non-ER oxycodone and brand name oxycodone tablets including OxyContin. Measured via MMEs, hydrocodone was shipped to WV in highest quantities until approximately 2012, when it was surpassed by non-ER oxycodone. OxyContin was shipped in third-highest quantities (measured via MMEs) after approximately 2007. The opioid products included in each category are available in Supplemental Tables 2–6.

**Fig. 3:**
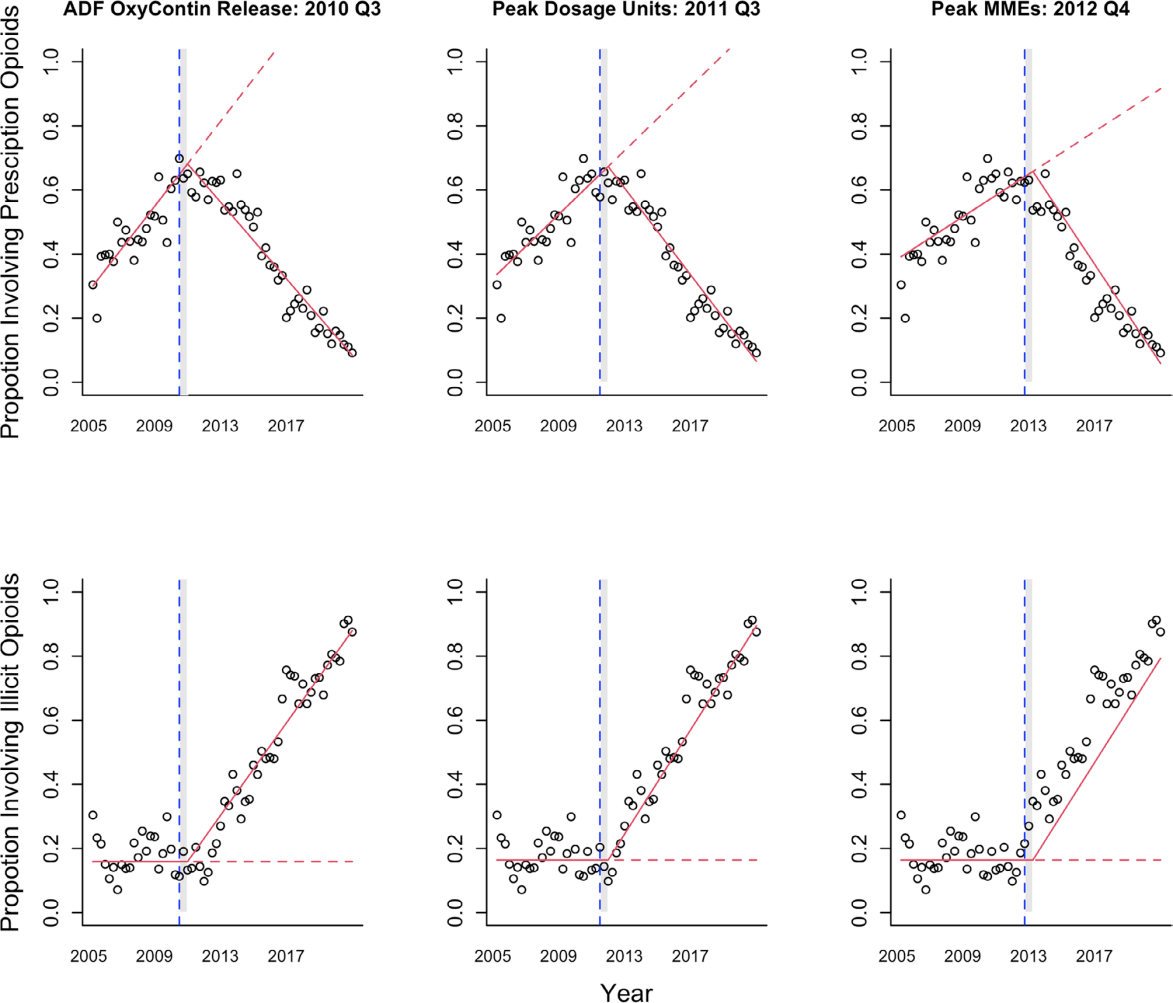
Graphical representation of interrupted time series analysis (ITSA)^a^ of the proportion quarterly opioid-involved overdose deaths involving prescription and illicit opioids^b^ ^a^ Upper and lower sub-figures represent ITSA of prescription and illicit opioid-involved overdose rates, respectively, while each column represents a unique ITSA intervention. Red lines represent estimated intervention impacts while dotted red lines represent estimated counterfactual (i.e., no intervention) trends. Blue dotted lines represent intervention start dates while grey shaded areas represent a six-month (two-quarter) transition period after which the intervention impact is theorized to have begun; a three-month (one-quarter) lag/transition period, which was modeled and tested separately, is not shown ^b^ Prescription opioid overdoses were defined as those associated with oxycodone or hydrocodone, while illicit opioid overdoses were defined as those involving heroin and synthetic opioids other than methadone, including fentanyl, fentanyl analogues, 4-anpp, and u-47700. Data are from the West Virginia Forensic Drug Database, which compiles data from the West Virginia Office of the Chief Medical Examiner

Graphical representation of each ITSA model with a 6-month lag and a corresponding counterfactual (i.e., no intervention) scenario is presented in Fig. 3; the blue dotted line indicates the intervention date of interest while the grey shaded area denotes a transition period of two quarters post-intervention. For all ITSA models, serial correlation was adequately controlled with an AR [1] ARIMA term (Tables 1 and 2). ITSA of illicit opioids was best modeled using only a ramp function, indicating there was no pre-intervention trend present (Table 2). For both prescription and illicit opioids, dosage units-informed ITSA models had lowest AIC, indicating best model fit; dosage-unit informed models with a 3-month lag had AIC of -189.2 and − 188.7 for prescription and illicit opioids, respectively, while models with a 6-month lag had AIC of -188.6 and − 189.4 for prescription and illicit opioids, respectively. For both 3- and 6-month lagged ITSA models of either prescription or illicit opioids, MME-informed models showed second lowest AIC and ADF OxyContin release third lowest AIC. For 3-month lagged models, dosage units-informed models were more than two AIC units lower than ADF release and peak MME-informed models, while MME- and ADF OxyContin-informed models were not meaningfully different (less than two AIC unit difference). For six-month lagged ITSA models, AIC difference between the three models was greater than two units for both prescription (peak dosage unit-informed model 3.1 and 10.3 units lower than ADF release and peak MME-informed models, respectively; Table 1) and illicit opioids (peak dosage units-informed model 3.8 and 8.7 units lower than ADF release and peak MME-informed models, respectively; Table 2), suggesting meaningful differences between each model’s performance. Intervention points informed using LOESS-identified changes in hydrocodone (2009 Q1 for both dosage units and MMEs; Supplementary Tables 7 and 8) and oxycodone (2014 Q1 for dosage units, 2014 Q2 for MMEs; Supplementary Tables 9 and 10) did not result in meaningfully better-fitting models (compared to the total opioid shipment informed models shown in Tables 1 and 2) for either prescription or illicit opioid overdose deaths.

**Table 1:** Interrupted time series results of opioid-involved overdoses in West Virginia involving prescription opioids.^a^

	3-Month Lag/Transition Period									6-Month Lag/Transition Period							
**Intervention: Oxycontin ADF released (2010 Q3)**									**Intervention: Oxycontin ADF released (2010 Q3)**								
	Parameter		Estimate		P-value		AIC			Parameter		Estimate		P-value		AIC	
	AR1 (𝜖)		0.36		0.002		-183.1			AR1 (𝜖)		0.32		0.007		-185.5	
	Intercept (𝛽_*0*_)		0.27		< 0.001					Intercept (𝛽_*0*_)		0.28		< 0.001			
	Time (𝛽_*1*_)		0.02		< 0.001					Time (𝛽_*1*_)		0.02		< 0.001			
	Ramp (𝛽_*2*_)		-0.03		< 0.001					Ramp (𝛽_*2*_)		-0.03		< 0.001			
**Intervention: Peak dosage units (2011 Q3)**									**Intervention: Peak dosage units (2011 Q3)**								
	Parameter		Estimate		P-value		AIC			Parameter		Estimate		P-value		AIC	
	AR1 (𝜖)		0.25		0.042		-189.2			AR1 (𝜖)		0.25		0.042		-188.6	
	Intercept (𝛽_*0*_)		0.31		< 0.001					Intercept (𝛽_*0*_)		0.32		< 0.001			
	Time (𝛽_*1*_)		0.01		< 0.001					Time (𝛽_*1*_)		0.01		< 0.001			
	Ramp (𝛽_*2*_)		-0.03		< 0.001					Ramp (𝛽_*2*_)		-0.03		< 0.001			
**Intervention: Peak MMEs (2012 Q4)**									**Intervention: Peak MMEs (2012 Q4)**								
	Parameter		Estimate		P-value		AIC			Parameter		Estimate		P-value		AIC	
	AR1 (𝜖)		0.38		0.001		-181.6			AR1 (𝜖)		0.43		< 0.001		-178.3	
	Intercept (𝛽_*0*_)		0.35		< 0.001					Intercept (𝛽_*0*_)		0.36		< 0.001			
	Time (𝛽_*1*_)		0.01		< 0.001					Time (𝛽_*1*_)		0.01		< 0.001			
	Ramp (𝛽_*2*_)		-0.03		< 0.001					Ramp (𝛽_*2*_)		-0.03		< 0.001			

^a^ Prescription opioid overdoses were defined as those associated with oxycodone or hydrocodone. Data are from the West Virginia Forensic Drug Database, which compiles data from the West Virginia Office of the Chief Medical Examiner

^b^ Akaike Information Criteria (AIC). A lower value is considered better model fit and a difference of more than two AIC units indicates a meaningfully better-fitting model

**Table 2:** Interrupted time series results of opioid-involved overdoses in West Virginia involving illicit opioids.^a^

	3-Month Lag/Transition Period									6-Month Lag/ Transition Period							
**Intervention: Oxycontin ADF released (2010 Q3)**									**Intervention: Oxycontin ADF released (2010 Q3)**								
	Parameter		Estimate		P-value		AIC			Parameter		Estimate		P-value		AIC	
	AR1 (𝜖)		0.60		< 0.001		-183.7			AR1 (𝜖)		0.57		< 0.001		-185.6	
	Intercept (𝛽_*0*_)		0.21		< 0.001					Intercept (𝛽_*0*_)		0.17		< 0.001			
	Ramp (𝛽_*2*_)		0.023		< 0.001					Ramp (𝛽_*2*_)		0.02		< 0.001			
**Intervention: Peak dosage units (2011 Q3)**									**Intervention: Peak dosage units (2011 Q3)**								
	Parameter		Estimate		P-value		AIC			Parameter		Estimate		P-value		AIC	
	AR1 (𝜖)		0.50		< 0.001		-188.7			AR1 (𝜖)		0.50		< 0.001		-189.4	
	Intercept (𝛽_*0*_)		0.18		< 0.001					Intercept (𝛽_*0*_)		0.18		< 0.001			
	Ramp (𝛽_*2*_)		0.02		< 0.001					Ramp (𝛽_*2*_)		0.02		< 0.001			
**Intervention: Peak MMEs (2012 Q4)**									**Intervention: Peak MMEs (2012 Q4)**								
	Parameter		Estimate		P-value		AIC			Parameter		Estimate		P-value		AIC	
	AR1 (𝜖)		0.11		< 0.001		-182.9			AR1 (𝜖)		0.67		< 0.001		-180.7	
	Intercept (𝛽_*0*_)		0.02		< 0.001					Intercept (𝛽_*0*_)		0.21		< 0.001			
	Ramp (𝛽_*2*_)		0.00		< 0.001					Ramp (𝛽_*2*_)		0.02		< 0.001			

^a^ Illicit opioid overdoses were defined as those involving heroin and synthetic opioids other than methadone, including fentanyl, fentanyl analogues, 4-anpp, and u-47,700. Data are from the West Virginia Forensic Drug Database, which compiles data from the West Virginia Office of the Chief Medical Examiner

^b^ Akaike Information Criteria (AIC). A lower value is considered better model fit and a difference of more than two AIC units indicates a meaningfully better-fitting model

## Discussion

Using ITSA, this study compared the impact of decreasing opioid tablet shipments, measured via dosage units (tablets) and MMEs, to the release of ADF OxyContin, which is generally acknowledged as the factor initiating a transition from prescription to illicit opioid use in the US [11, 12, 46]. Our findings suggest that in WV, overdose patterns began changing closer to the time when prescription opioid shipments (measured via dosage units) began decreasing in 2011 Q3. We also accounted for three- and six-month lag/transition periods (i.e., one and two quarter lag/transition periods, respectively) between shipment to observable effect on fatal overdose rates, indicating the impact observed via ITSA was a full 1.25 to 1.5 years after the ADF OxyContin release.

Changes in prescription opioid supplies have a measurable impact on use of both prescription and illicit opioids [2, 47–49]. Therefore, it is possible that decreasing prescription opioid supplies in WV during our study period contributed to increasing rates of overdoses involving illicit opioids. From 2006 to 2011, the WV opioid prescription dispensing rate was the highest of any US state [50]. However, WV opioid prescription rates, measured either via dosage units or MMEs, decreased more quickly than the US average during our study period [6, 50]. These drastic supply changes may explain our finding that the ITSA study based on overall opioid shipments to WV (measured via dosage units) was the preferred model for both prescription and illicit overdose rates in our study. Our data indicated that the majority of prescription opioid tablets shipped to the state were not OxyContin (Fig. 2); this is congruent with national data showing that the majority of oxycodone prescribed post-ADF OxyContin release was generic and therefore prone to misuse [13]. Thus, those who used OxyContin in WV likely had many other opioid options to begin using following the release of its ADF formulation. Moreover, those with OUD often prefer immediate release prescription opioid formulations as opposed to ER formulations [51]. This may be why the ADF OxyContin release did not fit our data well as a dosage unit-based intervention.

Soon after its release, the ADF OxyContin formulation was cited as a major contributor to subsequent increases in illicit opioid use and overdose [46]. However, analyses since the 2019 ARCOS data release by *The Washington Post* support our conclusion that changing supplies of other prescription opioids had a greater influence. For instance, Zhang and Guth assessed ARCOS, substance use data from The National Survey on Drug Use and Health (NSDUH), and opioid mortality data and found that the majority of OxyContin users in their sample transitioned to generic oxycodone after the ADF release [13]. The authors also noted that heroin mortality was highest in states with previously high generic oxycodone use and that illicit opioid overdose rates began increasing nearly two years after the ADF release. Our findings support and expand on those from Zhang and Guth by using ITSA, quarterly as opposed to annual data, and by including data on shipments of hydrocodone, a prescription opioid that was prescribed and misused at rates similar to OxyContin and oxycodone before the ADF OxyContin release [15–17].

While we used ARCOS data to assess prescription opioid supplies throughout our study period, analyses of other data sources support our conclusions. For example, NSDUH data indicate those using misusing OxyContin before its ADF release had 58% lower odds of heroin initiation than those using other prescription opioids [52]. Similarly, in a large sample of individuals screened for substance misuse, oxymorphone and buprenorphine use rates increased after the ADF OxyContin release while heroin use rates did not change significantly [10]. Another study using linked health insurance and National Death Index data did not find an overall effect on fatal and non-fatal overdoses; the study did find a small decrease in OxyContin overdose rates [9]. Finally, a 2020 US Food and Drug Administration (FDA) joint meeting of the Drug Safety and Risk Management Advisory Committee and the Anesthetic and Analgesic Drug Products Advisory Committee used multiple data sources to assess the national post-market impact of the ADF OxyContin release. Among the meeting’s findings were that while non-oral OxyContin use decreased after the ADF release, oral use increased significantly [36] and misuse of hydrocodone and other schedule II opioids increased significantly relative to OxyContin [37].

It is worthwhile to briefly discuss *why* decreasing prescription opioid supplies may have driven a mass transition to illicit opioid use. Previously published qualitative studies indicate that prescription opioid users who began using heroin during our study period did so because of the latter’s decreased cost relative to former, as well as ease of access to heroin [53]. While trends in national illicit opioid supplies are difficult to accurately quantify relative to prescription opioids, Customs and Border Patrol seizure data suggest that illicit heroin shipments to the US increased by more than 50% between 2012 and 2015; fentanyl seizures increased dramatically from 2015 to 2017 [54]. It has been suggested that these two trends (decreasing prescription opioids supplies and increasing illicit opioids supplies) are tied through supply-side economic principles; as restrictive or prohibitive constraints are placed on an addictive substance, availability of their illicit counterparts will increase to meet the pre-existing demand [8]. Of particular concern is the unpredictable variation in these illicit opioids’ potency and mixing with non-opioid substances, such as xylazine [55] and methamphetamine [24], which do not respond to opioid overdose reversal medications such as naloxone.

In addition to elucidating the transition from prescription to illicit opioid overdose in WV, this study expands on ITSA literature seeking to identify intervention dates using data that is related to, yet separate from, a time series of interest. Notably, Gilmour et al. used previously published survey data to identify a plausible ITSA start date of the Australian heroin shortage [42]. Similarly, Lopez Bernal et al. used a widely accepted definition for the beginning of an economic recession (the point at which gross domestic product growth rate is negative compared to previous quarter) to assess the impact of the late 2000’s financial crisis on suicide rates in Spain [56]. While these studies provided innovative approaches, the methods used to identify interventions were statistically descriptive. Using the inferential LOESS regression approach in our study, future studies might more accurately determine an intervention start date, when one is not easily defined, for ITSA study use.

This study has several strengths. For example, to the author’s knowledge, we are the first to demonstrate the feasibility of LOESS regression to determine a plausible intervention start date for use in an ITSA study of substance use data. As previous substance use research has identified the difficulty in studying interventions with no known start date [42], this is an interesting addition to current ITSA literature. Additionally, our study assessed medical examiner data from WV, which has a highly specific drug death investigation system relative to other states [57]. Our study also has several limitations. First, although ITSA is a powerful statistical study design useful in many situations in which a public health intervention has no control group, it remains an ecologic study design that cannot infer causality. Despite this, we believe the methodology in this study provides a robust approach towards strengthening evidence for a specific intervention’s impact since it uses ITSA to quantify the impact of several intervention points. Second, while changes in opioid tablet shipments occurred gradually over time, ITSA can only incorporate interventions at a single point in time. However, we tested each intervention at pre-specified, literature informed lag/transition periods (i.e., three and six-months) to account for the slow decline in opioid tablet distribution. Third, given urban/rural differences in prescription opioid misuse rates, our results may not be generalizable outside of WV, a largely rural state. Fourth, the medical examiner’s data used in this study relies on toxicology reports that cannot differentiate between formulations of the same drug. We therefore cannot assess which formulation of oxycodone (OxyContin or generic) or hydrocodone contributed to overdose rates throughout our study period. There is also the possibility for some missing or inaccurate data entries into the Forensic Drug Database, although this is believed to be minimal. Finally, ARCOS is limited to hydrocodone and oxycodone; while data suggest these were the primary opioids contributing to substance use disorder during the early years of the opioid epidemic in WV, other prescription opioids played a role.

## Conclusion

These results suggest that the transition from prescription to illicit opioid overdose in WV may have been affected to a greater extent by decreasing rates of prescription opioid shipments, as compared to the release of ADF OxyContin previously reported in studies of national data. The large quantity of hydrocodone shipped to WV (relative to OxyContin) might also have reduced the apparent impact on overdose deaths from the ADF OxyContin release. Future research should explore the impacts of supply-side changes in the availability of other prescription opioids in WV on not only drug-related deaths, but also on other outcomes, including substance use, substance use treatment, and related co-morbidities such as acute hepatitis C infection.

### Electronic supplementary material

Below is the link to the electronic supplementary material.

Supplementary Material 1: Supplemental Table 1: *Washington Post* ARCOS data variables used in the calculation of dosage units and MMEs. 
Supplemental Table 2: Hydrocodone products shown in Fig. 2, measured via total dosage units and morphine milligram equivalents (MMEs) shipped to West Virginia for 2006–2014. 
Supplemental Table 3. Generic extended-release oxycodone products shown in Fig. 2, measured via total dosage units and morphine milligram equivalents (MMEs) shipped to West Virginia for 2006–2014. 
Supplemental Table 4: Generic non-extended-release oxycodone products shown in Fig. 2, measured via total dosage units and morphine milligram equivalents (MMEs) shipped to West Virginia for 2006–2014. 
Supplemental Table 5: OxyContin products shown in Fig. 2, measured via total dosage units and morphine milligram equivalents (MMEs) shipped to West Virginia for 2006–2014. Bolded rows denoted abuse-deterrent formulations. 
Supplemental Table 6: Brand name oxycodone products (other than OxyContin) shown in Fig. 2, measured via total dosage units and morphine milligram equivalents (MMEs) shipped to West Virginia for 2006–2014. 
Supplemental Table 7: Interrupted time series results of opioid-involved overdoses in West Virginia involving prescription opioids; informed via LOESS regression of hydrocodone tablet shipments to WV. 
Supplemental Table 8: Interrupted time series results of opioid-involved overdoses in West Virginia involving illicit opioids; informed via LOESS regression of hydrocodone tablet shipments to WV. 
Supplemental Table 9: Interrupted time series results of opioid-involved overdoses in West Virginia involving prescription opioids; informed via LOESS regression of oxycodone (including OxyContin) tablet shipments to WV. 
Supplemental Table 10: Interrupted time series results of opioid-involved overdoses in West Virginia involving illicit opioids; informed via LOESS regression of oxycodone (including OxyContin) tablet shipments to WV.

## Data Availability

The ARCOS and West Virginia forensic drug datasets used in this study are available from the authors upon reasonable request.

## Abbreviations

ACF: Autocorrelation function
ADF: Abuse-deterrent formulation
AIC: Akaike Information Criteria
ARCOS: Automation of Reports and Consolidated Orders System
ARIMA: Autoregressive integrated moving average
DEA: Drug Enforcement Agency’s
ER: Extended release
FDD: Forensic drug database
LOESS: Locally estimated scatterplot smoothing
ITSA: Interrupted time series analysis
MME: Morphine milligram equivalent
NSDUH: National Survey on Drug Abuse and Health
OCME: Office of the Chief Medical Examiner
OUD: Opioid use disorder
PACF: Partial autocorrelation function
US: United States
WV: West Virginia
