# Supplementary material for: Comparing the effects of decreasing prescription opioid shipments and the release of an abuse deterrent OxyContin formulation on opioid overdose fatalities in WV: an interrupted time series study
Source: Subst Abuse Treat Prev Policy. 2024 Jan 4;19:4. doi: 10.1186/s13011-023-00587-2 (PMC10768117; doi:10.1186/s13011-023-00587-2)
Supplement: Supplementary file 1 — Supplementary Material 1: Supplemental Table 1: Washington Post ARCOS data variables used in the calculation of dosage units and MMEs. Supplemental Table 2: Hydrocodone products shown in Fig. 2, measured via total dosage units and morphine milligram equivalents (MMEs) shipped to West Virginia for 2006–2014. Supplemental Table 3. Generic extended-release oxycodone products shown in Fig. 2, measured via total dosage units and morphine milligram equivalents (MMEs) shipped to West Virginia for 2006–2014. Supplemental Table 4: Generic non-extended-release oxycodone products shown in Fig. 2, measured via total dosage units and morphine milligram equivalents (MMEs) shipped to West Virginia for 2006–2014. Supplemental Table 5: OxyContin products shown in Fig. 2, measured via total dosage units and morphine milligram equivalents (MMEs) shipped to West Virginia for 2006–2014. Bolded rows denoted abuse-deterrent formulations. Supplemental Table 6: Brand name oxycodone products (other than OxyContin) shown in Fig. 2, measured via total dosage units and morphine milligram equivalents (MMEs) shipped to West Virginia for 2006–2014. Supplemental Table 7: Interrupted time series results of opioid-involved overdoses in West Virginia involving prescription opioids; informed via LOESS regression of hydrocodone tablet shipments to WV. Supplemental Table 8: Interrupted time series results of opioid-involved overdoses in West Virginia involving illicit opioids; informed via LOESS regression of hydrocodone tablet shipments to WV. Supplemental Table 9: Interrupted time series results of opioid-involved overdoses in West Virginia involving prescription opioids; informed via LOESS regression of oxycodone (including OxyContin) tablet shipments to WV. Supplemental Table 10: Interrupted time series results of opioid-involved overdoses in West Virginia involving illicit opioids; informed via LOESS regression of oxycodone (including OxyContin) tablet shipments to WV. [file 13011_2023_587_MOESM1_ESM.docx]

**Supplemental Material**

**Supplemental Table 1.** *Washington Post* ARCOS data variables used in the calculation of dosage units and MMEs.

| **Variable** | **ARCOS Variable Name** | **ARCOS Data Dictionary Description** |
| --- | --- | --- |
| Quantity | DOSAGE_UNIT | DEA calculated field indicating number of pills, patches or lozenges, among others, shipped as part of the transaction. |
| Strength | dos_str | Strength of dose in milligrams. |
| Conversion Factor | MME_Conversion_Factor | Morphine Milligram Equivalent, or how the specific drug compares to a morphine equivalent. |

**Supplemental Table 2.** Hydrocodone products shown in Figure 2, measured via total dosage units and morphine milligram equivalents (MMEs) shipped to West Virginia for 2006-2014.

| **Product_Name** | **MME** | **Dosage_Units** |
| --- | --- | --- |
| ACET/HYDROCOD.BIT.,500MG&5MG/TAB | 15700 | 3140 |
| ACETA/HYDROCODONE.BIT - 750MG/7.5MG | 126825 | 16910 |
| ACETAMINOPHEN 750MG/ HYDROCODONE BIT | 11250 | 1500 |
| ANEXSIA 7.5MG TAB HYDROCODO.BIT ACET | 13500 | 1800 |
| ANEXSIA.HYDROCODONE.BITARTRATE.10MG/ | 1141000 | 114100 |
| CO-GESIC ACETA & HYDROCODO.BIT.,5MG | 2000 | 400 |
| HYCODAN 5MG/TABLET | 42000 | 8400 |
| HYCODAN TAB. / HYDROCODONE BIT. 5MG | 1000 | 200 |
| HYDRO-APAP 7.5MG HYDROCOD.BIT/325MG | 1350 | 180 |
| HYDROCOD. BIT 5MG TAB | 284360 | 56872 |
| HYDROCOD. BIT 7.5MG/ACETAMINOPHEN 75 | 73500 | 9800 |
| HYDROCOD. BIT5MG & ACET TAB | 13500 | 2700 |
| HYDROCOD.BIT 7.5 TAB | 15752250 | 2100300 |
| HYDROCOD.BIT. & ACET.;2.5MG/600MG/TA | 2180547.5 | 872219 |
| HYDROCOD.BIT. 5MG/HOMATROPINE.METHYL | 648000 | 129600 |
| HYDROCOD.BIT./ACET.,5MG & 500MG/TAB | 32500 | 6500 |
| HYDROCOD.BIT./ACET.,5MG & 500MG/TAB( | 7500 | 1500 |
| HYDROCOD.BIT./ACET.,7.5MG & 500MG/TA | 234375 | 31250 |
| HYDROCOD.BIT./ACET.,7.5MG & 750MG/TA | 27000 | 3600 |
| HYDROCOD.BIT./APAP;5MG & 500MG/TAB;B | 1500 | 300 |
| HYDROCOD.BIT./APAP;5MG&500MG/TAB;10X | 66500 | 13300 |
| HYDROCOD.BIT.& APAP,10MG/660MG/TAB | 50755000 | 5075500 |
| HYDROCOD.BIT.& APAP;7.5MG&750MG/TAB | 21600 | 2880 |
| HYDROCODO.BIT 10MG & ACETA TABLET | 11310000 | 1131000 |
| HYDROCODO.BIT 10MG&AC TAB | 38396000 | 3839600 |
| HYDROCODO.BIT 10MG&AC USP TAB | 252415000 | 25241500 |
| HYDROCODO.BIT 10MG&ACETAMINOPHEN USP | 63000 | 6300 |
| HYDROCODO.BIT 7.5MG TAB | 6658500 | 887800 |
| HYDROCODO.BIT.,7.5MG/APAP,750MG/TAB | 32850 | 4380 |
| HYDROCODO.BIT/APAP 7.5MG/750MG USP T | 157967250 | 21062300 |
| HYDROCODO.BIT5MG/AC TAB | 353500 | 70700 |
| HYDROCODONE & ACETA 5MG/500MG | 1500 | 300 |
| HYDROCODONE 10MG/APAP 325MG TABS | 6000 | 600 |
| HYDROCODONE 7.5MG;APAP 750MG TABS. | 4500 | 600 |
| HYDROCODONE BIT / ACETAMINOPHEN 10MG | 66464000 | 6646400 |
| HYDROCODONE BIT & ACETA 7.5MG/500MG | 38507775 | 5134370 |
| HYDROCODONE BIT & ACETAMINOPHEN 5MG/ | 1464000 | 292800 |
| HYDROCODONE BIT & ACETAMINOPHEN 7.5M | 1950000 | 260000 |
| HYDROCODONE BIT 5MG/ACETAMINOPHEN 50 | 168557040 | 33711408 |
| HYDROCODONE BIT. & ACETA 10MG/325MG | 696000 | 69600 |
| HYDROCODONE BIT. & ACETA 10MG/500MG | 1224000 | 122400 |
| HYDROCODONE BIT. & ACETA 5MG/325MG T | 21000 | 4200 |
| HYDROCODONE BIT. & ACETA 5MG/500MG T | 1639500 | 327900 |
| HYDROCODONE BIT. & ACETA. 7.5MG/750M | 479250 | 63900 |
| HYDROCODONE BIT. & IBUPROFEN 7.5MG/2 | 750 | 100 |
| HYDROCODONE BIT. & IBUPROPHEN 7.5MG/ | 1058250 | 141100 |
| HYDROCODONE BIT. 10MG/ACETAMINOPHEN | 915367000 | 91536700 |
| HYDROCODONE BIT. 5MG/ACETA. 325MG TA | 5500 | 1100 |
| HYDROCODONE BIT. 7.5MG/ACETAMINOPHEN | 59728931.25 | 7963857.5 |
| HYDROCODONE BIT.,7.5MG/ACET.500MG/TA | 900 | 120 |
| HYDROCODONE BIT./ACET.,7.5MG & 650MG | 75176250 | 10023500 |
| HYDROCODONE BIT./ACETA 10MG/325MG TA | 98090000 | 9809000 |
| HYDROCODONE BIT./ACETA 10MG/500MG US | 1302000 | 130200 |
| HYDROCODONE BIT./ACETA 5MG/325MG TAB | 11471000 | 2294200 |
| HYDROCODONE BIT./ACETA 5MG/325MG USP | 9500 | 1900 |
| HYDROCODONE BIT./ACETA 7.5MG/325MG T | 18059250 | 2407900 |
| HYDROCODONE BIT./ACETA 7.5MG/500MG 1 | 53250 | 7100 |
| HYDROCODONE BIT./ACETA. 10MG/325MG U | 2000 | 200 |
| HYDROCODONE BIT./ACETA. 7.5MG/325MG | 1500 | 200 |
| HYDROCODONE BIT./ACETA. TABLETS USP | 500 | 100 |
| HYDROCODONE BIT./ACETAM. TABS. 5MG/3 | 25500 | 5100 |
| HYDROCODONE BIT./ACETAMIN. TABS. 7.5 | 34500 | 4600 |
| HYDROCODONE BIT./ACETAMINOPHEN 10MG/ | 10000 | 1000 |
| HYDROCODONE BIT./ACETAMINOPHEN TABS. | 171344900 | 22863430 |
| HYDROCODONE BIT./APAP 10MG/325MG TAB | 41616000 | 4161600 |
| HYDROCODONE BIT./APAP 10MG/650MG TAB | 9368000 | 936800 |
| HYDROCODONE BIT./APAP 10MG/750MG TAB | 2000 | 200 |
| HYDROCODONE BIT./APAP 5MG/325MG TABL | 295400 | 59080 |
| HYDROCODONE BIT./APAP 7.5MG/325MG TA | 8160750 | 1088100 |
| HYDROCODONE BIT./APAP 7.5MG/650MG TA | 1120500 | 149400 |
| HYDROCODONE BIT./APAP 7.5MG/750MG 10 | 21750 | 2900 |
| HYDROCODONE BIT./IBUPROFEN;7.5MG & 2 | 9106500 | 1214200 |
| HYDROCODONE BIT.&ACETA 10MG/660MG TA | 91000 | 9100 |
| HYDROCODONE BIT.7.5MG/ACETAMINOPHEN | 418473750 | 55796500 |
| HYDROCODONE BIT/ ACETAMINOPHEN 5MG/5 | 47000 | 9400 |
| HYDROCODONE BIT/ACETA 10MG/325MG USP | 243305000 | 24330500 |
| HYDROCODONE BIT/ACETA 10MG/500MG USP | 463392000 | 46339200 |
| HYDROCODONE BIT/ACETA 5MG/325MG TABL | 136000 | 27200 |
| HYDROCODONE BIT/ACETA 5MG/325MG USP | 52575500 | 10515100 |
| HYDROCODONE BIT/ACETA 7.5MG/325MG US | 123987750 | 16531700 |
| HYDROCODONE BIT/ACETA 7.5MG/500MG US | 507744750 | 67699300 |
| HYDROCODONE BIT/ACETAMINOPHEN 10MG/3 | 29732500 | 2973250 |
| HYDROCODONE BIT/ACETAMINOPHEN 5MG/32 | 15350 | 3070 |
| HYDROCODONE BIT/ACETAMINOPHEN 5MG/50 | 507248500 | 101449700 |
| HYDROCODONE BIT/ACETAMINOPHEN 7.5MG/ | 19050 | 2540 |
| HYDROCODONE BIT/APAP 10MG/325MG TABL | 2455200 | 245520 |
| HYDROCODONE BIT/APAP 10MG/500MG TABL | 468000 | 46800 |
| HYDROCODONE BIT/APAP 5MG/325MG TABLE | 2266200 | 453240 |
| HYDROCODONE BIT/APAP 5MG/500MG TABLE | 594000 | 118800 |
| HYDROCODONE BIT/APAP 7.5MG/325MG TAB | 1612950 | 215060 |
| HYDROCODONE BIT/APAP 7.5MG/500MG TAB | 480600 | 64080 |
| HYDROCODONE BIT/HOMATROPINE METHYLBR | 880000 | 176000 |
| HYDROCODONE BIT/IBUPROFEN 5MG/200MG | 7500 | 1500 |
| HYDROCODONE BIT/IBUPROFEN 10MG/200MG | 2.00E+05 | 20000 |
| HYDROCODONE BIT/IBUPROFEN 2.5MG/200M | 3750 | 1500 |
| HYDROCODONE BIT/IBUPROFEN 7.5MG/200M | 2220750 | 296100 |
| HYDROCODONE BITARTARATE.2.5MG & ACET | 5364250 | 2145700 |
| HYDROCODONE BITARTRATE / ACETAMINOPH | 15000 | 1500 |
| HYDROCODONE BITARTRATE & ACETA 10MG/ | 12366000 | 1236600 |
| HYDROCODONE BITARTRATE & ACETA 5MG/3 | 7313000 | 1462600 |
| HYDROCODONE BITARTRATE & ACETA 7.5MG | 98076675 | 13076890 |
| HYDROCODONE BITARTRATE & ACETAMINOPH | 51201750 | 6747400 |
| HYDROCODONE BITARTRATE 10MG;ACETAMIN | 151000 | 15100 |
| HYDROCODONE BITARTRATE 10MG;GUAIFENE | 82000 | 8200 |
| HYDROCODONE BITARTRATE 10MG/ACETAMIN | 293905800 | 29390580 |
| HYDROCODONE BITARTRATE 5MG TAB | 1650 | 330 |
| HYDROCODONE BITARTRATE 5MG/ACETAMINO | 64066900 | 12813380 |
| HYDROCODONE BITARTRATE 7.5MG /ACETAM | 75600 | 10080 |
| HYDROCODONE BITARTRATE 7.5MG & ACETA | 29246250 | 3899500 |
| HYDROCODONE BITARTRATE 7.5MG/ACETAMI | 80486250 | 10731500 |
| HYDROCODONE BITARTRATE AND ACETA 10M | 23923000 | 2392300 |
| HYDROCODONE BITARTRATE AND ACETA 5MG | 21405000 | 4281000 |
| HYDROCODONE BITARTRATE AND ACETA 7.5 | 23453250 | 3127100 |
| HYDROCODONE BITARTRATE AND ACETAMINO | 7500 | 1000 |
| HYDROCODONE BITARTRATE/ APAP TABLETS | 20400 | 3080 |
| HYDROCODONE BITARTRATE/ APAP TABS US | 32250 | 4300 |
| HYDROCODONE BITARTRATE/ACETA 2.5MG/3 | 250 | 100 |
| HYDROCODONE BITARTRATE/ACETA 7.5MG/3 | 43074000 | 5743200 |
| HYDROCODONE BITARTRATE/APAP 10MG/500 | 73178000 | 7317800 |
| HYDROCODONE BITARTRATE/IBUPROFEN 5MG | 8500 | 1700 |
| HYDROCODONE BITARTRATE/IBUPROFEN 7.5 | 39000 | 5200 |
| HYDROCODONE BITARTRATE/IBUPROPHEN 7. | 21000 | 2800 |
| HYDROCODONE.BIT & ACETA 10MG & 500M | 159945500 | 15994550 |
| HYDROCODONE.BIT 7.5MG/ACETAMINAOPHEN | 21000 | 2800 |
| HYDROCODONE.BIT. & ACETA 5MG & 500M | 68102000 | 13620400 |
| HYDROCODONE.BIT. & ACETA, 10MG & 500 | 18500 | 1850 |
| HYDROCODONE.BIT./ACET.,10MG & 325MG/ | 369246500 | 36924650 |
| HYDROCODONE.BIT/IBUPROFEN 10MG/200MG | 525000 | 52500 |
| HYDROCODONE.BITARTRATE 10MG & ACETAM | 497000 | 49700 |
| HYDROCODONE.BITARTRATE 10MG/APAP 650 | 18674000 | 1867400 |
| HYDROCODONE.BITARTRATE 2.5MG/IBUPROF | 3750 | 1500 |
| HYDROCODONE.BITARTRATE 7.5MG/APAP 65 | 1872750 | 249700 |
| HYDROCODONE.BITARTRATE 7.5MG/APAP 75 | 7008750 | 934500 |
| HYDROCODONE.BITARTRATE/ACETA 10MG/50 | 1000 | 100 |
| HYDROCODONE.BITARTRATE/IBUPROFEN 7.5 | 22134750 | 2951300 |
| HYDROCODONE.BITRATRATE.10MG/ACETAMIN | 13430000 | 1343000 |
| HYDROCODONE/ACETAMINOPHEN 5MG/500MG | 13909500 | 2781900 |
| HYDROCODONE/APAP - 5MG/500MG TABLET | 3000 | 600 |
| HYDROCODONE/APAP 10MG/325MG - 10MG H | 4200 | 420 |
| HYDROCODONE/APAP 10MG/500MG TABLETS | 12000 | 1200 |
| HYDROCODONE/APAP 5MG/500MG TABLETS | 594400 | 118880 |
| HYDROCODONE/APAP 5MG/500MG (LORTAB G | 4000 | 800 |
| HYDROCODONE/APAP 7.5MG/200MG TABS. | 1972500 | 263000 |
| HYDROCODONE/IBUPROFEN 5MG/200MG TABL | 127000 | 25400 |
| HYDROCODONE/IBUPROFEN 7.5MG/200MG TA | 22771500 | 3036200 |
| IBUDONE HYDROCODONE BIT./IBUPROFEN 1 | 73800 | 7380 |
| IBUDONE/HYDROCODONE BIT./IBUPROFEN | 59900 | 11980 |
| IBUDONE/HYDROCODONE BIT./IBUPROFEN T | 85500 | 10400 |
| LORCET HYD.BIT10MG/ACET650MG TAB | 3543000 | 354300 |
| LORCET HYD.BIT10MG/ACET650MG TAB (4 | 4000 | 400 |
| LORCET PLUS HYDROCODO.BIT 7.5/ACET65 | 919500 | 122600 |
| LORCET+ HYDROCODO.BIT7.5MG/ACET650MG | 75000 | 10000 |
| LORTAB 10 TAB HYDROCODO.BIT & AC | 908000 | 90800 |
| LORTAB 10MG/500MG/TAB,HYDROCOD.BIT.& | 8922000 | 892200 |
| LORTAB 5 HYDROCODO/AC TAB | 996500 | 199300 |
| LORTAB 7.5 HYD.BIT/AC TAB | 718500 | 95800 |
| LORTAB 7.5MG HYDROCODONE.BIT / 500MG | 4953000 | 660400 |
| MAXIDONE;HYDROCODONE BIT./ACET.,10MG | 45000 | 4500 |
| NORCO 5/325;5MG HYDROCOD.BIT. & 325M | 149500 | 29900 |
| NORCO HYDROCODO.BIT./ACET.,10MG & 32 | 2009000 | 200900 |
| NORCO TAB - HYDROCODONE BIT/ACETA 10 | 455000 | 45500 |
| NORCO TAB - HYDROCODONE BIT/ACETA 7. | 207750 | 27700 |
| NORCO;7.5MG HYDROCOD.BIT.+ 325MG ACE | 379500 | 50600 |
| P-V-TUSSIN TABS;5MG HYDROCODO.BIT. & | 17500 | 3500 |
| PNEUMOTUSSIN TABS;2.5MG HYDROCOD.BIT | 1000 | 400 |
| REPREXAIN - HYDROCODONE BIT./IBUPROP | 985175 | 127050 |
| REPREXAIN - HYDROCODONE.BIT. 5MG/IBU | 3600 | 720 |
| REPREXAIN (HYDROCODONE BITARTRATE/IB | 7000 | 700 |
| TUSSEND;5MG HYDROCOD.BIT./TAB | 1000 | 200 |
| TUSSIGON 5MG HYD.BIT&HOMA MBR 1.5MG/ | 1151000 | 230200 |
| TUSSO HC HYDRO.BIT.10MG;GUAIF.1200MG | 2000 | 200 |
| VICODIN ES HYDROCODONE BITRATE/ACETA | 1053750 | 140500 |
| VICODIN ES TABLETS 7.5MG HYDROCODONE | 5898750 | 786500 |
| VICODIN HP HYDROCODONE BITARTRATE/AC | 1418000 | 141800 |
| VICODIN HP TABLETS 10MG HYDROCODONE. | 2366000 | 236600 |
| VICODIN HYDROCODONE BITARTRATE/ACETA | 797000 | 159400 |
| VICODIN TABLETS 5MG HYDROCODONE.BIT | 756235 | 151247 |
| VICOPROFEN TABLETS 7.5MG HYDROCODONE | 1156500 | 154200 |
| VICOPROFEN;7.5MG HYDROCOD.BIT.& 200M | 1800 | 240 |
| XODOL - HYDROCODONE.BIT 10MG & ACETA | 929200 | 92920 |
| XODOL 5MG HYDRO. BIT.;300MG ACET. TA | 354000 | 70800 |
| XODOL 7.5/300MG HYDROCODONE.BIT. & A | 108750 | 14500 |
| XODOL 7.5MG HYDROCODONE BIT. ;300MG | 678750 | 90500 |
| XODOL HYDROCODONE.BITARTRATE 5MG/ACE | 167000 | 33400 |
| XODOL- HYDROCODONE BIT/APAP 10MG/300 | 17000 | 1700 |
| XODOL- HYDROCODONE BIT/APAP 5MG/300M | 1500 | 300 |
| XODOL- HYDROCODONE BIT/APAP 7.5MG/30 | 8250 | 1100 |
| XODOL;10MG HYDRO.BIT.;300MG ACET. TA | 2507000 | 250700 |
| XPECT-HC/HYDRO.BIT.5MG/GUAIFENESIN 6 | 35300 | 7060 |
| ZTUSS/HYDRO.BIT.5MG/PSEUDOE.HC30MG/G | 4000 | 800 |
| ZYDONE TABS;10MG HYDROCOD.BIT.& 400M | 4503000 | 450300 |
| ZYDONE TABS;5MG HYDROCOD.BIT.& 400MG | 211500 | 42300 |
| ZYDONE TABS;7.5MG HYDROCOD.BIT.& 400 | 1719750 | 229300 |

**Supplemental Table 3.** Generic extended-release oxycodone products shown in Figure 2, measured via total dosage units and morphine milligram equivalents (MMEs) shipped to West Virginia for 2006-2014.

| **Product_Name** | **MME** | **Dosage_Units** |
| --- | --- | --- |
| OXYCOD.HCL ER TABS;10MG/TAB | 1582500 | 105500 |
| OXYCOD.HCL ER TABS;20MG/TAB | 4455000 | 148500 |
| OXYCOD.HCL ER TABS;40MG/TAB | 7254000 | 120900 |
| OXYCOD.HCL ER TABS;80MG/TAB | 1848000 | 15400 |
| OXYCODONE 10MG ER TABLETS | 90000 | 6000 |
| OXYCODONE 20MG ER TABLETS | 279000 | 9300 |
| OXYCODONE 40MG ER TABLETS | 792000 | 13200 |
| OXYCODONE 80MG ER TABLETS | 936000 | 7800 |
| OXYCODONE HCL 10MG ER TABLET | 6000 | 400 |
| OXYCODONE HCL 10MG ER TABLETS | 13500 | 900 |
| OXYCODONE HCL 20 MG ER TABLETS | 33000 | 1100 |
| OXYCODONE HCL 20MG ER TABLETS | 1755000 | 58500 |
| OXYCODONE HCL 40MG ER TABLET | 30000 | 500 |
| OXYCODONE HCL 40MG ER TABLETS | 54000 | 900 |
| OXYCODONE HCL 80MG ER TABLET | 12000 | 100 |
| OXYCODONE HCL 80MG ER TABLETS | 96000 | 800 |
| OXYCODONE HYDRCHLORIDE 40MG EXTENDED | 34134000 | 568900 |
| OXYCODONE HYDROCHLORIDE - ER - 80MG/ | 37704000 | 314200 |
| OXYCODONE HYDROCHLORIDE EXTENDED REL | 4671000 | 311400 |
| OXYCODONE HYDROCHLORIDE EXTENDED-REL | 18843000 | 628100 |
| OXYCODONE.HCL ER 10MG TABS | 2146500 | 143100 |
| OXYCODONE.HCL ER 20MG TABS | 8127000 | 270900 |
| OXYCODONE.HCL ER 40MG TABS | 16116000 | 268600 |
| OXYCODONE.HCL ER 80MG TABS | 11940000 | 99500 |
| OXYCODONE.HCL ER TABS;80MG/TAB | 756000 | 6300 |

**Supplemental Table 4.** Generic non-extended-release oxycodone products shown in Figure 2, measured via total dosage units and morphine milligram equivalents (MMEs) shipped to West Virginia for 2006-2014.

| **Product_Name** | **MME** | **Dosage_Units** |
| --- | --- | --- |
| ACET/OXYCOD.HCL;500MG&5.35MG/TAB | 817747.5 | 101900 |
| OXYCOD.HCL IR TABS;5MG/TAB | 303000 | 40400 |
| OXYCOD.HCL/APAP TABS;7.5MG & 500MG/T | 549000 | 48800 |
| OXYCOD.HCL/APAP;10MG & 650MG/TAB,BOT | 3139500 | 209300 |
| OXYCOD.HCL/APAP;2.5MG & 325MG/TAB;BO | 117000 | 31200 |
| OXYCODO.HCL 5.35MG/TAB | 5034885 | 627400 |
| OXYCODONE & ACETAMINOPHEN 10MG/325MG | 129000 | 8600 |
| OXYCODONE AND ACETA 7.5MG/325MG USP | 6996375 | 621900 |
| OXYCODONE AND ACETA 10MG/325MG TABLE | 25446000 | 1696400 |
| OXYCODONE AND ACETA 7.5MG / 500MG TA | 19280250 | 1713800 |
| OXYCODONE AND ACETAMINOPHEN 10MG/325 | 307500 | 20500 |
| OXYCODONE AND ACETAMINOPHEN 7.5MG/32 | 2176875 | 193500 |
| OXYCODONE HCI 20 MG TABLETS USP | 73062000 | 2435400 |
| OXYCODONE HCI 10MG TABLETS USP | 92359500 | 6157300 |
| OXYCODONE HCI 5MG TABLETS | 23661750 | 3154900 |
| OXYCODONE HCI/APAP 5/325MG TABLETS | 18000 | 2400 |
| OXYCODONE HCL & ACETA 10MG/325MG USP | 789000 | 52600 |
| OXYCODONE HCL & ACETA 5MG/325MG USP | 102000 | 13600 |
| OXYCODONE HCL & ACETA 7.5MG/325MG US | 279000 | 24800 |
| OXYCODONE HCL & IBUPROPHEN 5MG/400MG | 158250 | 21100 |
| OXYCODONE HCL & NIACIN 7.5MG/30MG US | 18000 | 1600 |
| OXYCODONE HCL 10MG CR TABLETS | 427500 | 28500 |
| OXYCODONE HCL 10MG IR TABLET | 25068000 | 1671200 |
| OXYCODONE HCL 10MG IR TABS | 802500 | 53500 |
| OXYCODONE HCL 10MG TABLET USP; 10 X | 4500 | 300 |
| OXYCODONE HCL 10MG TABLET USP; 100 T | 15000 | 1000 |
| OXYCODONE HCL 10MG TABLETS | 96000 | 6400 |
| OXYCODONE HCL 10MG TABS | 4939500 | 329300 |
| OXYCODONE HCL 10MG USP TABLETS | 127500 | 8500 |
| OXYCODONE HCL 15MG IR TABLET | 164250 | 7300 |
| OXYCODONE HCL 15MG IR TABS | 13578750 | 603500 |
| OXYCODONE HCL 15MG TABLET USP; 100 T | 132750 | 5900 |
| OXYCODONE HCL 15MG TABLETS | 28235250 | 1254900 |
| OXYCODONE HCL 15MG TABLETS, 100 CT | 179892000 | 7995200 |
| OXYCODONE HCL 15MG TABLETS, 100CT | 272250 | 12100 |
| OXYCODONE HCL 15MG TABLETS, USP | 35113500 | 1560600 |
| OXYCODONE HCL 15MG USP TABLETS | 251417250 | 11174100 |
| OXYCODONE HCL 20MG CR TABLETS | 21000 | 700 |
| OXYCODONE HCL 20MG IR BLEND TABS | 984000 | 32800 |
| OXYCODONE HCL 20MG IR TABLET | 19824000 | 660800 |
| OXYCODONE HCL 20MG TABLET USP; 100 T | 111000 | 3700 |
| OXYCODONE HCL 20MG TABLETS | 534000 | 17800 |
| OXYCODONE HCL 20MG USP TABLETS | 234000 | 7800 |
| OXYCODONE HCL 30MG IR TABLET | 153000 | 3400 |
| OXYCODONE HCL 30MG IR TABS | 20614500 | 458100 |
| OXYCODONE HCL 30MG TABLET USP; 100 T | 679500 | 15100 |
| OXYCODONE HCL 30MG TABLETS | 45859500 | 1019100 |
| OXYCODONE HCL 30MG TABLETS, 100 CT | 368158500 | 8181300 |
| OXYCODONE HCL 30MG TABLETS, 100CT | 261000 | 5800 |
| OXYCODONE HCL 30MG TABLETS, USP | 56281500 | 1250700 |
| OXYCODONE HCL 30MG USP TABLETS | 531652500 | 11814500 |
| OXYCODONE HCL 40MG CR TABLETS | 60000 | 1000 |
| OXYCODONE HCL 40MG TABS | 43866000 | 731100 |
| OXYCODONE HCL 5MG IR TABLET | 240750 | 32100 |
| OXYCODONE HCL 5MG TABLET USP; 10 X 1 | 111750 | 14900 |
| OXYCODONE HCL 5MG TABLET USP; 100 TA | 42750 | 5700 |
| OXYCODONE HCL 5MG TABLETS | 4566750 | 608900 |
| OXYCODONE HCL 5MG TABLETS, 100CT | 42000 | 5600 |
| OXYCODONE HCL 5MG TABLETS, USP | 9674250 | 1289900 |
| OXYCODONE HCL 5MG USP TABLETS | 21885750 | 2918100 |
| OXYCODONE HCL 5MG/IBUPROFEN 400MG TA | 237750 | 31700 |
| OXYCODONE HCL 80MG CR TABLETS | 144000 | 1200 |
| OXYCODONE HCL 80MG TABS | 39948000 | 332900 |
| OXYCODONE HCL AND ASPIRIN 4.8355MG/3 | 195112.425 | 26900 |
| OXYCODONE HCL USP & IBUPROFEN 5MG/40 | 109500 | 14600 |
| OXYCODONE HCL/ACETAMINOPHEN 10MG/325 | 447124500 | 29808300 |
| OXYCODONE HCL/ACETAMINOPHEN 10MG/650 | 8418000 | 561200 |
| OXYCODONE HCL/ACETAMINOPHEN 2.5MG/32 | 223500 | 59600 |
| OXYCODONE HCL/ACETAMINOPHEN 5MG/325M | 403299000 | 53773200 |
| OXYCODONE HCL/ACETAMINOPHEN 7.5MG/32 | 108936000 | 9683200 |
| OXYCODONE HCL/ACETAMINOPHEN 7.5MG/50 | 2665125 | 236900 |
| OXYCODONE HCL/ACETAMINOPHEN TABLET U | 4084500 | 544600 |
| OXYCODONE HCL/ACETAMINOPHEN TABS 10M | 146827500 | 9788500 |
| OXYCODONE HCL/ACETAMINOPHEN TABS 5MG | 33074250 | 4409900 |
| OXYCODONE HCL/ACETAMINOPHEN TABS. 10 | 24906000 | 1660400 |
| OXYCODONE HCL/ACETAMINOPHEN TABS. 7. | 2730375 | 242700 |
| OXYCODONE HCL/ASPIRIN 4.8355/325MG T | 163198.125 | 22500 |
| OXYCODONE HYDROCHLORIDE 10MG TABLETS | 453000 | 30200 |
| OXYCODONE HYDROCHLORIDE 15MG TABLET | 69750 | 3100 |
| OXYCODONE HYDROCHLORIDE 15MG TABLETS | 281826000 | 12525600 |
| OXYCODONE HYDROCHLORIDE 20MG TABLETS | 1782000 | 59400 |
| OXYCODONE HYDROCHLORIDE 20MG TABS. | 6174000 | 205800 |
| OXYCODONE HYDROCHLORIDE 30MG TABLET | 710356500 | 15785700 |
| OXYCODONE HYDROCHLORIDE 40MG TABLETS | 3660000 | 61000 |
| OXYCODONE HYDROCHLORIDE 40MG TABS. | 11334000 | 188900 |
| OXYCODONE HYDROCHLORIDE 5MG TABS USP | 797250 | 106300 |
| OXYCODONE HYDROCHLORIDE 5MG&ACETAMIN | 15054000 | 2007200 |
| OXYCODONE HYDROCHLORIDE 80MG TABLETS | 4368000 | 36400 |
| OXYCODONE HYDROCHLORIDE 80MG TABS. | 13320000 | 111000 |
| OXYCODONE HYDROCHLORIDE CONTROLLED R | 144000 | 2800 |
| OXYCODONE HYDROCHLORIDE CR 20MG TABL | 20343000 | 678100 |
| OXYCODONE HYDROCHLORIDE TABLETS 5MG | 81765000 | 10902000 |
| OXYCODONE HYDROCHLORIDE TABLETS USP | 152565750 | 6780700 |
| OXYCODONE HYDROCHLORIDE TABS. 10MG | 1006500 | 67100 |
| OXYCODONE HYDROCHLORIDE USP 30MG TAB | 232398000 | 5164400 |
| OXYCODONE.HCL 10MG / APAP 650MG TABL | 89059500 | 5937300 |
| OXYCODONE.HCL 5MG IR (10 X 10 BLISTE | 22500 | 3000 |
| OXYCODONE.HCL 5MG IR TAB | 2973750 | 396500 |
| OXYCODONE.HCL/APAP 10MG/325MG TABS | 166764000 | 11117600 |
| OXYCODONE.HCL/APAP 7.5MG/325MG TABS | 37197000 | 3306400 |
| OXYCODONE.HCL/APAP TABLETS, 7.5MG/50 | 17239500 | 1532400 |
| OXYCODONE/APAP 5MG/325MG TABS. | 10125 | 1350 |

**Supplemental Table 5.** OxyContin products shown in Figure 2, measured via total dosage units and morphine milligram equivalents (MMEs) shipped to West Virginia for 2006-2014. Bolded rows denoted abuse-deterrent formulations.

| **Product_Name** | **MME** | **Dosage_Units** |
| --- | --- | --- |
| OXYCONTIN - 10MG OXYCODONE.HCL CONTR | 14522400 | 968160 |
| OXYCONTIN - 40MG OXYCODONE.HCL CONTR | 232012800 | 3866880 |
| OXYCONTIN - 80MG OXYCODONE.HCL CONTR | 290431200 | 2420260 |
| OXYCONTIN (OXYCODONE.HCL) CONTROLLED | 76090950 | 2536365 |
| **OXYCONTIN 10MG OXYCODONE HCL CR TABL** | **19325400** | **1288360** |
| OXYCONTIN 15MG CONTROLLED RELEASE OX | 1512000 | 67200 |
| **OXYCONTIN 15MG OXYCODONE HCL CR TABL** | **6472800** | **287680** |
| **OXYCONTIN 20MG OXYCODONE HCL CR TABL** | **62755800** | **2091860** |
| OXYCONTIN 30MG COTROLLED RELEASE OXY | 18346500 | 407700 |
| **OXYCONTIN 30MG OXYCODONE HCL CR TABL** | **38507400** | **855720** |
| **OXYCONTIN 40MG OXYCODONE HCL CR TABL** | **123261600** | **2054360** |
| OXYCONTIN 60MG COTROLLED RELEASE OXY | 48069000 | 534100 |
| **OXYCONTIN 60MG OXYCODONE HCL CR TABL** | **68443200** | **760480** |
| **OXYCONTIN 80MG OXYCODONE HCL CR TABL** | **189686400** | **1580720** |

**Supplemental Table 6.** Brand name oxycodone products (other than OxyContin) shown in Figure 2, measured via total dosage units and morphine milligram equivalents (MMEs) shipped to West Virginia for 2006-2014.

| **Product_Name** | **MME** | **Dosage_Units** |
| --- | --- | --- |
| COMBUNOX - 5MG / 400MG OXYCODONE.HCL | 1065000 | 142000 |
| ENDOCET - 10MG OXYCODONE.HCL/325MG A | 243274500 | 16218300 |
| ENDOCET - 7.5MG OXYCODONE.HCL/325MG | 35419500 | 3148400 |
| ENDOCET OXYCODO HCL5MG&AC TAB | 34386000 | 4584800 |
| ENDOCET TABS - 10MG OXYCODONE.HCL & | 140868000 | 9391200 |
| ENDOCET TABS;7.5MG OXYCODONE.HCL & 5 | 18250875 | 1622300 |
| ENDODAN OXYCODONE & ASP, USP 4.8355M | 501199.575 | 69100 |
| MAGNACET 10MG/400MG OXYCODONE HCL/AC | 885000 | 59000 |
| MAGNACET 5MG/400MG OXYCODONE HCL/ACE | 3000 | 400 |
| MAGNACET 7.5MG/400MG OXYCODONE HCL/A | 43875 | 3900 |
| MAGNACET TM /OXYCODONE 10MG;ACET.400 | 361500 | 24100 |
| MAGNACET TM OXYCODONE 2.5MG ACET. TA | 3750 | 1000 |
| MAGNACET TM OXYCODONE 5MG;ACETA. 400 | 27000 | 3600 |
| MAGNACET TM OXYCODONE 7.5MG;ACET.400 | 65250 | 5800 |
| MAGNACET-OXYCODONE HCL/APAP 10MG/400 | 1500 | 100 |
| PERCOCET (OXYCODONE HCL/ACETA) 5MG/3 | 2325000 | 310000 |
| PERCOCET TABLETS 10MG OXYCODONE HCL/ | 8898000 | 593200 |
| PERCOCET TABLETS OXYCODONE HCL 7.5MG | 929250 | 82600 |
| PERCODAN - 4.8355MG OXYCODONE.HCL & | 455504.1 | 62800 |
| PERLOOX OXYCODONE.HCL 5MG/ACETAMINOP | 12750 | 1700 |
| PERLOXX OXYCODONE.HCL 10MG/ACETAMINO | 40500 | 2700 |
| PERLOXX OXYCODONE.HCL 7.5MG/ACETAMIN | 9000 | 800 |
| PRIMALEV OXYCODONE HCL 10MG & ACETA | 109500 | 7300 |
| PRIMALEV OXYCODONE HCL 2.5MG & ACETA | 375 | 100 |
| PRIMALEV OXYCODONE HCL 5MG & ACETA 3 | 27000 | 3600 |
| PRIMALEV OXYCODONE HCL 7.5MG & ACETA | 4500 | 400 |
| PRIMLEV - OXYCODONE HCI/ACETAMINOPHE | 25875 | 1800 |
| ROXICET - OXYCODONE.HCL & ACETA 5MG/ | 18892500 | 2519000 |
| ROXICODONE - 15MG OXYCODONE HCL TABL | 69750 | 3100 |
| ROXICODONE - 30MG OXYCODONE HCL TABL | 715500 | 15900 |
| ROXICODONE (OXYCODONE HCI);30MG;100 | 1944000 | 43200 |
| ROXICODONE (OXYCODONE HCL) 15MG TABS | 2772000 | 123200 |
| ROXICODONE (OXYCODONE HCL) 30MG TABS | 7137000 | 158600 |
| ROXICODONE (OXYCODONE HCL) 5MG TABS. | 381750 | 50900 |
| ROXICODONE 5MG TAB | 17250 | 2300 |
| ROXICODONE TABS;15MG OXYCODONE.HCL/T | 182250 | 8100 |
| ROXICODONE TABS;30MG OXYCODONE.HCL/T | 369000 | 8200 |
| ROXICODONE TABS.;(OXYCODONE HCI);15M | 1599750 | 71100 |
| ROXICODONE TABS.(OXYCODONE HCI);5MG; | 92250 | 12300 |
| ROXILOX OXYCO HCL 5MG &ACE500MG TAB | 3714750 | 495300 |
| XARTEMIS XR - OXYCODONE HCL/ACETA 7. | 142875 | 12700 |
| XOLOX - OXYCODONE HCL/ACETA 10MG/50 | 55500 | 3700 |

**Supplementary Table 7.** Interrupted time series results of opioid-involved overdoses in West Virginia involving prescription opioids; informed via LOESS regression of hydrocodone tablet shipments to WV.^a^

|  | **Intervention: Peak hydrocodone dosage units/MMEs (both 2009 Q1)** | | | | | | |  | |
| --- | --- | --- | --- | --- | --- | --- | --- | --- | --- |
|  | **3-Month Lag/Transition** | | | | | | |  | |
|  | Parameter |  | Estimate |  | P-value |  | AIC ^b^ | |  |
|  | AR1 (𝜖) |  | 0.63 |  | <0.001 |  | -169.1 |  | |
|  | Intercept (𝛽*_0_*) |  | 0.26 |  | <0.001 |  |  |  | |
|  | Time (𝛽*_1_*) |  | 0.02 |  | <0.001 |  |  |  | |
|  | Ramp (𝛽*_2_*) |  | -0.03 |  | <0.001 |  |  |  | |
|  |  |  |  |  |  |  |  |  | |
|  | **6-Month Lag/Transition** | | | | | | | | |
|  | Parameter |  | Estimate |  | P-value |  | AIC |  | |
|  | AR1 (𝜖) |  | 0.58 |  | <0.001 |  | -169.9 |  | |
|  | Intercept (𝛽*_0_*) |  | 0.26 |  | <0.001 |  |  |  | |
|  | Time (𝛽*_1_*) |  | 0.02 |  | <0.001 |  |  |  | |
|  | Ramp (𝛽*_2_*) |  | -0.03 |  | <0.001 |  |  |  | |

^a^ Prescription opioid overdoses were defined as those associated with oxycodone or hydrocodone. Data are from the West Virginia Forensic Drug Database, which compiles data from the West Virginia Office of the Chief Medical Examiner.

^b^ Akaike Information Criteria (AIC). A lower value is considered better model fit and a difference of more than two AIC units indicates a meaningfully better-fitting model.

**Supplementary Table 8.** Interrupted time series results of opioid-involved overdoses in West Virginia involving illicit opioids; informed via LOESS regression of hydrocodone tablet shipments to WV.^a^

|  | **Intervention: Peak hydrocodone dosage units/MMEs (both 2009 Q1)** | | | | | | |  |
| --- | --- | --- | --- | --- | --- | --- | --- | --- |
|  | **3-Month Lag/Transition** | | | | | | |  |
|  | Parameter |  | Estimate |  | P-value |  | AIC ^b^ |  |
|  | AR1 (𝜖) |  | 0.74 |  | <0.001 |  | -178.0 |  |
|  | Intercept (𝛽*_0_*) |  | 0.14 |  | <0.001 |  |  |  |
|  | Ramp (𝛽*_2_*) |  | 0.02 |  | <0.001 |  |  |  |
|  |  |  |  |  |  |  |  |  |
|  | **6-Month Lag/Transition** | | | | | | | |
|  | Parameter |  | Estimate |  | P-value |  | AIC |  |
|  | AR1 (𝜖) |  | 0.71 |  | <0.001 |  | -178.1 |  |
|  | Intercept (𝛽*_0_*) |  | 0.15 |  | <0.001 |  |  |  |
|  | Ramp (𝛽*_2_*) |  | 0.02 |  | <0.001 |  |  |  |

^a^ Illicit opioid overdoses were defined as those involving heroin and synthetic opioids other than methadone, including fentanyl, fentanyl analogues, 4-anpp, and u-47700. Data are from the West Virginia Forensic Drug Database, which compiles data from the West Virginia Office of the Chief Medical Examiner.

^b^ Akaike Information Criteria (AIC). A lower value is considered better model fit and a difference of more than two AIC units indicates a meaningfully better-fitting model.

**Supplementary Table 9.** Interrupted time series results of opioid-involved overdoses in West Virginia involving prescription opioids; informed via LOESS regression of oxycodone (including OxyContin) tablet shipments to WV.^a^

|  | **3-Month Lag** | | | | | | |  |
| --- | --- | --- | --- | --- | --- | --- | --- | --- |
|  | **Intervention: Peak oxycodone dosage units (2014 Q1)** | | | | | | |  |
|  | Parameter |  | Estimate |  | P-value |  | AIC |  |
|  | AR1 (𝜖) |  | 0.61 |  | <0.001 |  | -173.7 |  |
|  | Intercept (𝛽*_0_*) |  | 0.39 |  | <0.001 |  |  |  |
|  | Time (𝛽*_1_*) |  | 0.01 |  | 0.002 |  |  |  |
|  | Ramp (𝛽*_2_*) |  | -0.03 |  | <0.001 |  |  |  |
|  |  |  |  |  |  |  |  |  |
|  | **Intervention: Peak oxycodone MMEs (2014 Q2)** | | | | | | |  |
|  | Parameter |  | Estimate |  | P-value |  | AIC |  |
|  | AR1 (𝜖) |  | 0.65 |  | <0.001 |  | -169.0 |  |
|  | Intercept (𝛽*_0_*) |  | 0.40 |  | <0.001 |  |  |  |
|  | Time (𝛽*_1_*) |  | 0.01 |  | 0.009 |  |  |  |
|  | Ramp (𝛽*_2_*) |  | -0.03 |  | <0.001 |  |  |  |
|  |  |  |  |  |  |  |  |  |
|  | **6-Month Lag** | | | | | | | |
|  | **Intervention: Peak oxycodone dosage units (2014 Q1)** | | | | | | |  |
|  | Parameter |  | Estimate |  | P-value |  | AIC |  |
|  | AR1 (𝜖) |  | 0.65 |  | <0.001 |  | -169.0 |  |
|  | Intercept (𝛽*_0_*) |  | 0.40 |  | <0.001 |  |  |  |
|  | Time (𝛽*_1_*) |  | 0.01 |  | 0.009 |  |  |  |
|  | Ramp (𝛽*_2_*) |  | -0.03 |  | <0.001 |  |  |  |
|  |  |  |  |  |  |  |  |  |
|  | **Intervention: Peak oxycodone MMEs (2014 Q2)** | | | | | | |  |
|  | Parameter |  | Estimate |  | P-value |  | AIC |  |
|  | AR1 (𝜖) |  | 0.68 |  | <0.001 |  | -167.5 |  |
|  | Intercept (𝛽*_0_*) |  | 0.40 |  | <0.001 |  |  |  |
|  | Time (𝛽*_1_*) |  | 0.00 |  | 0.030 |  |  |  |
|  | Ramp (𝛽*_2_*) |  | -0.03 |  | <0.001 |  |  |  |

^a^ Prescription opioid overdoses were defined as those associated with oxycodone or hydrocodone. Data are from the West Virginia Forensic Drug Database, which compiles data from the West Virginia Office of the Chief Medical Examiner.

^b^ Akaike Information Criteria (AIC). A lower value is considered better model fit and a difference of more than two AIC units indicates a meaningfully better-fitting model.

**Supplementary Table 10.** Interrupted time series results of opioid-involved overdoses in West Virginia involving illicit opioids; informed via LOESS regression of oxycodone (including OxyContin) tablet shipments to WV.^a^

|  | **3-Month Lag** | | | | | | |  |
| --- | --- | --- | --- | --- | --- | --- | --- | --- |
|  | **Intervention: Peak oxycodone dosage units (2014 Q1)** | | | | | | |  |
|  | Parameter |  | Estimate |  | P-value |  | AIC |  |
|  | AR1 (𝜖) |  | 0.79 |  | <0.001 |  | -177.4 |  |
|  | Intercept (𝛽*_0_*) |  | 0.24 |  | <0.001 |  |  |  |
|  | Ramp (𝛽*_2_*) |  | 0.03 |  | <0.001 |  |  |  |
|  |  |  |  |  |  |  |  |  |
|  | **Intervention: Peak oxycodone MMEs (2014 Q2)** | | | | | | |  |
|  | Parameter |  | Estimate |  | P-value |  | AIC |  |
|  | AR1 (𝜖) |  | 0.81 |  | <0.001 |  | -176.0 |  |
|  | Intercept (𝛽*_0_*) |  | 0.25 |  | <0.001 |  |  |  |
|  | Ramp (𝛽*_2_*) |  | 0.03 |  | <0.001 |  |  |  |
|  |  |  |  |  |  |  |  |  |
|  | **6-Month Lag** | | | | | | | |
|  | **Intervention: Peak oxycodone dosage units (2014 Q1)** | | | | | | |  |
|  | Parameter |  | Estimate |  | P-value |  | AIC |  |
|  | AR1 (𝜖) |  | 0.81 |  | <0.001 |  | -176.0 |  |
|  | Intercept (𝛽*_0_*) |  | 0.25 |  | <0.001 |  |  |  |
|  | Ramp (𝛽*_2_*) |  | 0.03 |  | <0.001 |  |  |  |
|  |  |  |  |  |  |  |  |  |
|  | **Intervention: Peak oxycodone MMEs (2014 Q2)** | | | | | | |  |
|  | Parameter |  | Estimate |  | P-value |  | AIC |  |
|  | AR1 (𝜖) |  | 0.83 |  | <0.001 |  | -175.9 |  |
|  | Intercept (𝛽*_0_*) |  | 0.26 |  | <0.001 |  |  |  |
|  | Ramp (𝛽*_2_*) |  | 0.03 |  | <0.001 |  |  |  |
|  |  |  |  |  |  |  |  |  |

^a^ Illicit opioid overdoses were defined as those involving heroin and synthetic opioids other than methadone, including fentanyl, fentanyl analogues, 4-anpp, and u-47700. Data are from the West Virginia Forensic Drug Database, which compiles data from the West Virginia Office of the Chief Medical Examiner.

^b^ Akaike Information Criteria (AIC). A lower value is considered better model fit and a difference of more than two AIC units indicates a meaningfully better-fitting model.
